# Clinical inertia on insulin treatment intensification in type 2 diabetes mellitus patients of a tertiary public diabetes center with limited pharmacologic armamentarium from an upper-middle income country

**DOI:** 10.1186/s13098-018-0382-x

**Published:** 2018-10-29

**Authors:** Marcelo Alves Alvarenga, William Ricardo Komatsu, Joao Roberto de Sa, Antonio Roberto Chacra, Sergio Atala Dib

**Affiliations:** Department of Medicine, Endocrinology Division, Diabetes Center, UNIFESP (Federal University of São Paulo), Rua Estado de Israel, 639 Vila Clementino, São Paulo, SP CEP 04022-001 Brazil

## Abstract

**Background:**

Clinical inertia is related to the difficulty of achieving and maintaining optimal glycemic control. It has been extensively studied the delay of the period to insulin introduction in type 2 diabetes mellitus (T2DM) patients. This study aims to evaluate clinical inertia of insulin treatment intensification in a group of T2DM patients followed at a tertiary public Diabetes Center with limited pharmacologic armamentarium (Metformin, Sulphonylurea and Human Insulin).

**Methods:**

This is a real life retrospective record based study with T2DM patients. Demographic, clinical and laboratory characteristics were reviewed. Clinical inertia was considered when the patients did not achieve the individualized glycemic goals and there were no changes on insulin daily dose in the period.

**Results:**

We studied 323 T2DM patients on insulin therapy (plus Metformin and or Sulphonylurea) for a period of 2 years. The insulin daily dose did not change in the period and the glycated hemoglobin (A1c) ranged from 8.8 + 1.8% to 8.7 ± 1.7% (basal vs 1st year; ns) and to 8.5 ± 1.8% (basal vs 2nd year; p = 0.035). The clinical inertia prevalence was 65.8% (basal), 61.9% (after 1 year) and 58.2% (after 2 years; basal vs 1st year vs 2nd year; ns). In a subgroup of 100 patients, we also studied the first 2 years after insulin introduction. The insulin daily dose ranged from 0.22 ± 0.12 to 0.32 ± 0.24 IU/kg of body weight/day (basal vs 1st year; p < 0.001) and to 0.39 ± 0.26 IU/kg of body weight/day (basal vs 2nd year; p < 0.05). The A1c ranged from 9.6 + 2.1% to 8.6 + 2% (basal vs 1st year; p < 0.001) and to 8.7 + 1.7% (1st year vs 2nd year; ns). The clinical inertia prevalence was 78.5% (at the moment of insulin therapy introduction), 56.2% (after 1 year; p = 0.001) and 62.2% (after 2 years; ns).

**Conclusion:**

Clinical inertia prevalence ranged from 56.2 to 78.5% at different moments of the insulin therapy (first 2 years and long term) of T2DM patients followed at a tertiary public Diabetes Center from an upper-middle income country with limited pharmacologic armamentarium.

**Electronic supplementary material:**

The online version of this article (10.1186/s13098-018-0382-x) contains supplementary material, which is available to authorized users.

## Background

There are significant evidences that hyperglycemia is associated to both microvascular and macrovascular complications and it is one of the risk factors on the clinical course of cardiovascular diseases [1, 2]. Nevertheless, uncontrolled glycemia is a global problem and most of type 2 diabetes mellitus (T2DM) patients often do not reach recommended glycemic targets in daily clinical practice [3–6, 27, 39, 40].

Diabetes guidelines typically advocate a target glycated hemoglobin (A1c) value of 6.5 or 7.0% but highlight that glycemic management must be individualized (individually adjusted A1c), considering a less stringent goal (A1c between 7 and 8%) for the patients with severe hypoglycemia risk, elderly, limited life expectancy or extensive comorbid conditions [7–10].

Insulin should be considered for patients with T2DM when noninsulin antihyperglycemic therapy fails to achieve target glycemic control or when a patient has symptomatic hyperglycemia [12, 13]. While the difficulty of maintaining the desired A1c level over time is related to both lifestyle and type of prescribed medication, it derives primarily from the progressive decline in beta cell function, with the need of insulin as the natural result of this temporal process [8, 9, 11, 34].

Another important aspect related to the difficulty of achieving and maintaining optimal glycemic control is the clinical inertia, defined as the failure to initiate or intensify therapy when indicated [14–16, 31–34]. It also may apply to the failure of physicians to stop or reduce therapy no longer needed [37]. Clinical inertia for insulin introduction has been extensively studied in T2DM patients [14–16, 26–28, 31–36]. It is well documented in Western countries, however similar data in low-middle income countries are lacking [51–53], specifically in the real world public healthcare system context where there are restrictions on antihyperglycemic therapy availability.

Therefore, the objective of this study was to evaluate the clinical inertia of insulin treatment intensification in a group of T2DM patients followed at a tertiary public Diabetes Center with limited pharmacologic armamentarium.

## Methods

### Study design

This is a real life retrospective record based study conducted at Federal University of São Paulo, Diabetes Center, which is a tertiary, teaching and research center and it is part of the public healthcare system, the Brazilian Unified Health System *(Sistema Único de Saúde, SUS)*. The total number of physicians/day is 9 (3 preceptors and 6 residents) that see 30 patients in 4 h. No private insurance is available. Metformin, Sulphonylurea and Human Insulin (NPH Insulin and Regular) are provided free of charge.

**Fig. 1:**
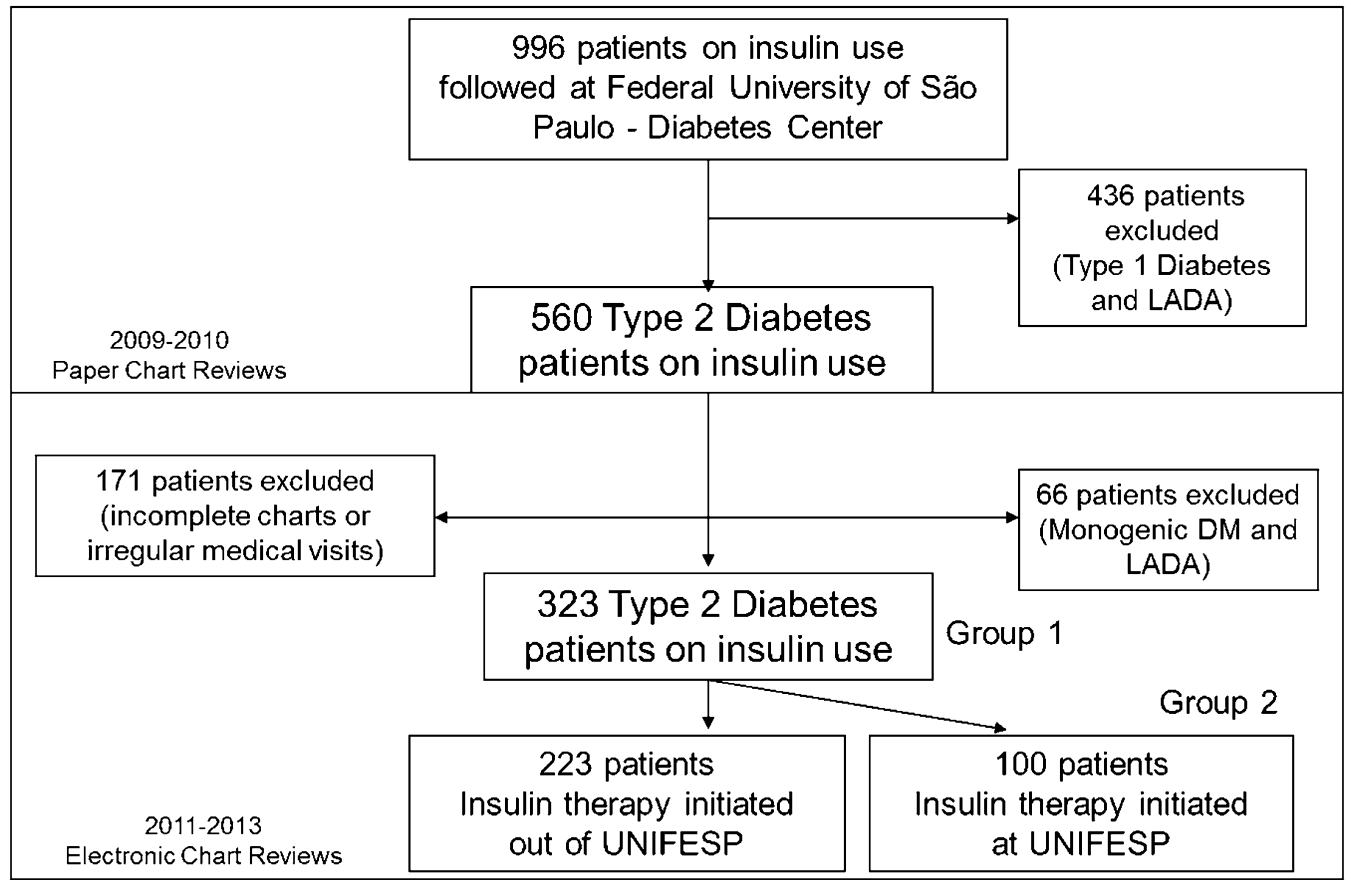
Study flowchart

We reviewed 996 charts of T2DM patients on insulin use, with or without oral antihyperglycemic therapy. Type 1 diabetes mellitus (T1DM), Latent Autoimmune Diabetes of the Adult (LADA), Monogenic Diabetes or other specific types of diabetes mellitus (DM) were exclusion criteria. Only the patients who had at least 1 visit to doctor per year (3 visits in 2 years period) were considered for the study (inclusion criteria). We excluded the patients with irregular medical appointments (less than 1 visit per year) or incomplete charts (absence of clinical or laboratory data), lasting 323 T2DM patients (32.4% of the initial sample of potential patients) on insulin therapy, eligible for the study (Fig. 1).

The study population had their demographic, clinical and laboratory characteristics reviewed, focusing on individually glycemic goals, insulin therapy (initiation, intensification, dose adjustment, weight gain and hypoglycemia), renal function, lipid profile and lipid therapy.

We called Group 1 the total studied population (T2DM patients on insulin therapy followed at UNIFESP Diabetes Center, independently if the insulin therapy was initiated at UNIFESP or the patient has been referred already on insulin using) and Group 2 is a subgroup of Group 1, whose insulin therapy was initiated at UNIFESP Diabetes Center. For the evaluation of clinical inertia during insulin treatment intensification in T2DM, we studied the last of 2 years of insulin therapy in Group 1 (323 patients) and the first 2 years after insulin therapy introduction in Group 2 (100 patients).

### Follow-up variables studied

The following demographic and clinical data were studied: gender, age, duration of clinical diabetes, criteria for insulin therapy introduction, time between diabetes diagnosis and onset of insulin treatment, body-mass index [BMI (kg/m2)], prevalence of chronic diabetes complications and severe hypoglycemia, number of medical visits per year, prevalence of self-blood glucose monitoring, oral anti-hyperglycemic therapy, insulin therapy [type of insulin, daily dose (IU/kg/day), number and time of daily injections] and dyslipidemia therapy. The laboratory data evaluated were: Glycated Hemoglobin (A1c), using high-performance liquid chromatography (HPLC) method (nv: 4.0–5.6%); lipid profile (total cholesterol, LDL-cholesterol, HDL-cholesterol and triglycerides) and renal function (estimated creatinine clearance rate), using Cockcroft–Gault formula.

Chronic diabetes complications (neuropathy, nephropathy, retinopathy and macroangiopathy) were diagnosed in according to ADA (American Diabetes Association) criteria [10] and severe hypoglycemia was defined as glycemia < 45 mg/dL or the occurrence of stupor, seizure or unconsciousness, during which someone else’s help was required [46].

Clinical inertia during insulin intensification was considered when there was no therapy adjustment (no statistically significant differences on insulin daily dose within 1 year interval period) [26, 27, 38, 40] in the patients out of the A1c targets (A1c < 8% for the patients with advanced diabetes complications or 65 years old or more and A1c < 7% for the others) [7–10].

### Statistical analysis

Statistical analysis was performed with the Statistic Package for Social Sciences (SPSS, Inc. Chicago II, USA) version 20.0 for Windows and Minitab 17.

Data were expressed as mean + SD values, median, maximum and minimum levels (quantitative data) and absolute or relative frequency (qualitative data). The significance level was set at p < 0.05 with a 95% confidence interval.

Generalized Estimating Equations (GEE) with normal, Poisson and binomial distribution for the longitudinal data analysis and Bonferroni’s Multiple Comparisons to compare groups were performed. Simple and Multiple Logistic Regression analysis were performed to evaluate the variables associated with achieving glycemic goals (individually adjusted A1c).

Chi Square test for association was performed to analyze and to compare the whole cohort A1c mean and the A1c subgroups (the percentage of patients in each A1c interval: < 7%, 7–8%, 8–9%, 9–10% and > 10%) for both Group 1 and Group 2. The significance level was set at p < 0.05 with a 95% confidence interval.

## Results

The Group 1 (whose clinical inertia during a period of 2 years of insulin therapy was studied) had 60.7% females, age of 65.8 ± 10 years, clinical diabetes duration of 18.6 ± 7.5 years and 10.6 ± 6.6 years of insulin therapy. The prevalence of diabetes chronic complications was: retinopathy 44%, nephropathy 63.1%, neuropathy 42.1% and macroangiopathy 43%. Systemic arterial hypertension and dyslipidemia were respectively presented in 96.0% and 88.2% patients. The most common insulin regimen was NPH insulin alone (41.8%) followed by basal-bolus (NPH plus Regular insulin) (39.3%). The most frequency of daily insulin injections were three times a day in 33.4% (before breakfast, before lunch and bedtime) and twice a day in 30.3% (before breakfast and bedtime). The most frequent oral anti-hyperglycemic therapy associated to insulin was Metformin (MET) (38.4%), followed by the association of MET + sulphonylurea (SU) (16.1%) and 24.1% of the patients did not use anti-hyperglycemic therapy, but only insulin. The total insulin daily dose ranged from 0.64 ± 0.4 to 0.67 ± 0.43 IU/kg of body weight/day in the first year and to 0.67 ± 0.41 IU/kg of body weight/day in the second year (basal vs. first and second year; ns) (Table 1).

**Table 1:** Clinical and laboratory evolution during the last 2 years of insulin therapy follow up (Group 1)

	Age—years	Duration of diabetes—years	Duration of insulin use—years					
Mean (sd)	65.8 (10)	18.6 (7.5)	10.6 (6.6)					
Median (min.; max.)	66 (37; 96)	17 (4; 42)	10 (1; 29)					

Variable	Basal	1 year after	2 years after	Comparison^a^	p	95% CI	
						Lower	Upper
Body-mass index, kg/m^2^				Basal—1 year after	0.148	− 0.67	0.07
Mean (sd)	28.7 (5.1)	29.1 (5)	29.4 (5.2)	Basal—2 years after	*0.002*	− 0.90	− 0.16
				1 year after–2 years after	0.386	− 0.59	0.13
Oral diabetes therapy				Basal—1 year after	0.876	− 2.00	6.00
n/Total (%)	219/289 (75.8)	205/279 (73.5)	189/267 (70.8)	Basal—2 years after	*0.029*	0.00	9.00
				1 year after—2 years after	*0.342*	− 1.00	7.00
Insulin daily dose, units/kg of body weight							
Mean (sd)	0.64 (0.4)	0.67 (0.43)	0.67 (0.41)		0.571		
Glycated hemoglobin / A1c				Basal—1 year after	> 0.999	− 0.15	0.29
Mean (sd)	8.8 (1.8)	8.7 (1.7)	8.5 (1.8)	Basal—2 years after	*0.003*	0.08	0.52
				1 year after—2 years after	*0.035*	0.01	0.45
Diabetes controlled (adjusted A1c), %				Basal—1 year after	0.509	− 10.00	3.00
n/Total (%)	108/316 (34.2)	118/310 (38.1)	128/306 (41.8)	Basal—2 years after	*0.016*	− 14.00	− 1.00
				1 year after–2 years after	0.479	− 10.00	3.00
Total cholesterol, mg/dL				Basal—1 year after	> 0.999	− 3.55	7.82
Mean (sd)	168.7 (44.2)	165.5 (43)	172.9 (43.5)	Basal—2 years after	0.101	− 10.95	0.66
				1 year after–2 years after	0.008	− 13.11	− 1.45
LDL cholesterol, mg/dL				Basal—1 year after	> 0.999	− 3.41	6.34
Mean (sd)	92.7 (36.5)	90.2 (34.8)	95.9 (37.3)	Basal—2 years after	0.117	− 9.28	0.69
				1 year after–2 years after	0.017	− 10.74	− 0.78
HDL cholesterol, mg/dL				Basal—1 year after	*0.026*	− 2.60	− 0.12
Mean (sd)	45.5 (12.1)	46.6 (12.8)	48.7 (14.6)	Basal—2 years after	*< 0.001*	− 4.80	− 2.27
				1 year after–2 years after	*< 0.001*	− 3.44	− 0.90
Triglycerides, mg/dL							
Mean (sd)	155.6 (111.8)	145.1 (89.8)	144 (80)		0.157		

GEE with normal distribution

^a^Bonferroni’s multiple comparisons

When we studied the glucose self-monitoring daily frequency in our population, we observed that 34.9% of patients did not monitor domiciliary glycemia values to adjust insulin dose, 26.6% and 22.7% measured respectively, once or twice a day. Reviewing the frequency of medical visits, we observed that 53.1% of the patients had 2 or more visits per year.

**Fig. 2:**
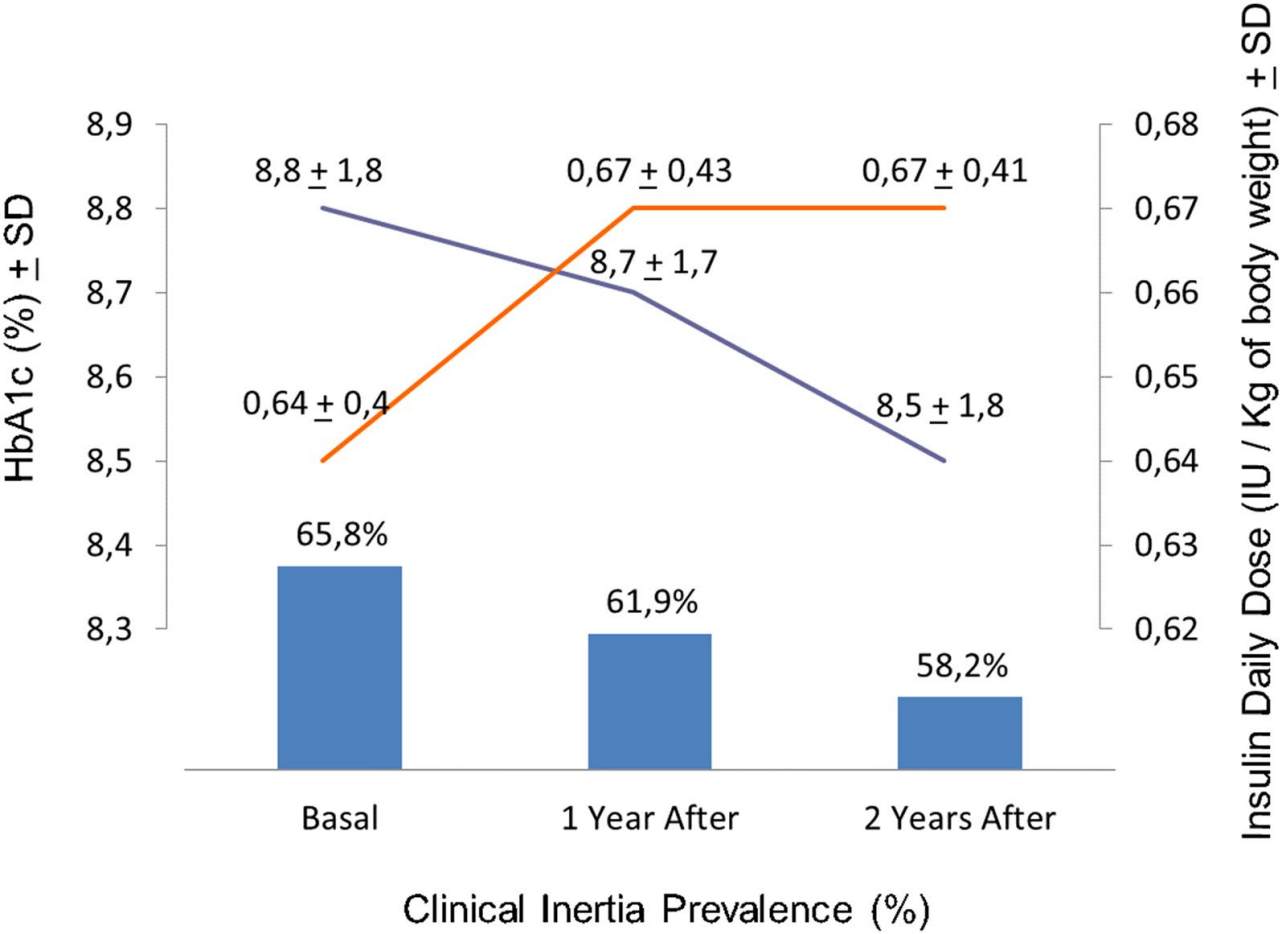
HbA1c, insulin daily dose and clinical inertia prevalence in Group 1 (323 Type 2 diabetes mellitus patients) during 2 years of follow up

The A1c ranged from 8.8 ± 1.8% to 8.7 ± 1.7% (ns) in the first year and to 8.5 ± 1.8% (p = 0.035) in the second year. We also analyzed A1c variation by subgroups (the percentage of patients in each A1c interval: < 7%, 7–8%, 8–9%, 9–10% and > 10%) and we did not find statistical differences (p = 0.257) among the groups in the studied periods (basal, first year and second year) (Additional file 1: Figure S1). The percentage of patients who achieved the A1c target was respectively 34.2%, 38.1% (basal vs. first year; ns) and 41.8% (first vs. second year; ns) (Fig. 2). Therefore, the clinical inertia was respectively 65.8%, 61.9% and 58.2% (basal vs. first year vs. second year; ns). Most patients were overweight, the BMI ranged from 28.7 ± 5.1 kg/m^2^ to 29.1 ± 5 kg/m^2^ (basal vs. first year; ns) and to 29.4 + 5.2 kg/m^2^ in the second year (first vs. second year; ns) and the occurrence of severe hypoglycemia in the period was 3.1%. Reviewing the lipid profile, HDL cholesterol has increased (p < 0.001) and LDL cholesterol has decreased (p = 0.017) in the period and the number of patients with lipid therapy did not change (ns).

In a simple logistic regression analysis of the Group 1, we observed that the age, male gender and the presence of macroangiopathy, nephropathy or retinopathy were the main isolated variables associated with achieving glycemic goals (individually adjusted A1c). In a multiple logistic regression analysis, the age, male gender and the presence of retinopathy were associated with achieving A1c targets. Each 1-year of age has increased 4% in the chance of achieving glycemic goals (p = 0.004), male gender had 74% more chances of diabetes control (p = 0.040) and the presence of retinopathy had 65% more chances (p = 0.060) (Table 2).

**Table 2:** Logistic regression analysis—variables associated with achieving glycemic goals (individually adjusted A1c) during the last 2 years of insulin therapy follow up (Group 1)

Variable	Diabetes control		Total	OR_Crude_	95% CI		p	OR_Adjusted_	95% CI		p
	No	Yes			Lower	Upper			Lower	Upper	
Age, years				1.06	1.03	1.08	*<* *0.001*	1.04	1.01	1.07	*0.004*
Mean (sd)	64 (9.9)	69 (9.4)	65.8 (10)								
Median (min.; max.)	65 (37; 96)	70 (38; 92)	66 (37; 96)								
Gender, n (%)											
Female	134 (68.7)	61 (31.3)	195	1.00				1.00			
Male	72 (56.7)	55 (43.3)	127	1.68	1.06	2.67	*0.029*	1.74	1.03	2.94	*0.040*
Duration of diabetes, years				1.00	0.97	1.03	0.899				
Mean (sd)	18.6 (7.5)	18.7 (7.6)	18.6 (7.5)								
Median (min.; max.)	17 (4; 42)	17 (4; 41)	17 (4; 42)								
Duration of insulin use, years				0.97	0.91	1.03	0.294				
Mean (sd)	11.1 (6.7)	9.7 (6.3)	10.6 (6.6)								
Median (min.; max.)	10 (1; 29)	9 (1; 29)	10 (1; 29)								
Systemic arterial hypertension, n (%)											
No	8 (66.7)	4 (33.3)	12	1.00							
Yes	198 (64.1)	111 (35.9)	309	1.12	0.33	3.81	0.854				
Dyslipidemia, n (%)											
No	22 (61.1)	14 (38.9)	36	1.00							
Yes	183 (64.2)	102 (35.8)	285	0.88	0.43	1.79	0.715				
Neuropathy, n (%)											
No	168 (65.4)	89 (34.6)	257	1.00							
Yes	33 (58.9)	23 (41.1)	56	1.32	0.73	2.38	0.363				
Nephropathy, n (%)											
No	175 (67.8)	83 (32.2)	258	1.00							
Yes	24 (46.2)	28 (53.8)	52	2.46	1.34	4.50	*0.004*				
Retinopathy, n (%)											
No	119 (69.6)	52 (30.4)	171	1.00				1.00			
Yes	81 (57.4)	60 (42.6)	141	1.70	1.06	2.70	*0.027*	1.65	0.98	2.78	*0.060*
Macroangiopathy, n (%)											
No	124 (70.9)	51 (29.1)	175	1.00							
Yes	77 (55.8)	61 (44.2)	138	1.93	1.21	3.08	*0.006*				
Insulin daily dose, units/kg of body weight				0.51	0.26	1.01	0.052				
Mean (sd)	0.68 (0.43)	0.58 (0.33)	0.65 (0.4)								
Median (min.; max.)	0.61 (0.11; 3.33)	0.5 (0.07; 1.48)	0.58 (0.07; 3.33)								
Glucose self monitoring daily											
No	70 (64.2)	39 (35.8)	109	1.00							
Yes	123 (64.4)	68 (35.6)	191	0.99	0.61	1.62	0.975				

Logistic regression

The Group 2 (whose clinical inertia during the first 2 years after insulin therapy introduction was also studied) had 56% females, age of 67.1 ± 10 years and clinical diabetes duration of 17.3 ± 6.3 years. The prevalence of diabetes chronic complications in this group was: retinopathy 8%, nephropathy 35%, neuropathy 20% and macroangiopathy 29%. Systemic arterial hypertension and dyslipidemia were respectively presented in 88% and 84% of the patients. The mean interval between DM diagnosis and the onset of insulin therapy in this subgroup was 10.7 + 6.4 years. The justification for initiating the insulin therapy was: the persistence of fasting blood glucose levels > 200 mg/dL (41%), A1c > 9% (35%), the presence of catabolism (17%) and any contraindicating factor to the oral agents (e.g. renal dysfunction). The insulin regime prescribed was basal insulin (NPH), 85% once a day (73% bed time and 12% before breakfast) and 15% twice a day (before breakfast and bedtime). By the time of insulin introduction, the most frequent oral anti-hyperglycemic therapy was the association of MET plus SU (59.6%). After insulin introduction, 63% of patients had no changing on oral anti-hyperglycemic therapy, 24% had SU withdrawal, 8% MET withdrawal and 2% both MET and SU withdrawal. The insulin daily dose ranged from 0.22 ± 0.12 to 0.32 ± 0.24 IU/kg of body weight/day (p < 0.001) in the first year and to 0.39 ± 0.26 IU/kg of body weight/day (p < 0.05) in the second year (Table 3). The A1c ranged from 9.6 ± 2.1% to 8.6 ± 2.0% (basal vs. first year; p < 0.001) and to 8.7 ± 1.7% in the second year (first vs. second year; ns). We also analyzed A1c variation by subgroups (the percentage of patients in each A1c interval: < 7%, 7–8%, 8–9%, 9–10% and > 10%) and we did not find statistical differences (p = 0.290) among the groups in the studied periods (first year and second year) (Additional file 2: Figure S2). The percentage of patients who achieved glycemic goals in this period was respectively 21.5%, 43.8% (p = 0.001) and 37.8% (ns). Therefore, the clinical inertia prevalence was 78.5% (at the moment of insulin therapy introduction), 56.2% (after 1 year; p = 0.001) and 62.2% (after 2 years; ns). The occurrence of severe hypoglycemia was 1% during the period. The BMI ranged from 28.4 ± 6.4 kg/m^2^ to 29.3 ± 5.5 kg/m^2^ (p = 0.070) in the first year and to 29 ± 5.3 kg/m^2^ in the second year (ns). Reviewing the lipid profile, both triglycerides (p = 0.023) and total cholesterol (p < 0.001) have improved in the first 2 years of insulin use and the number of patients with lipid therapy has increased (p = 0.05).

**Table 3:** Clinical and laboratory evolution during the first 2 years of insulin therapy follow up (Group 2)

	Age—years	Duration of diabetes—years	Duration of insulin use—years				
Mean (sd)	67.1 (10)	17.3 (6.3)	10.7 (6.4)				
Median (min.; max.)	67 (37; 96)	16.5 (4; 33)	10 (1; 29)				

Variable	Insulin introduction	1 year after insulin introduction	2 years after insulin introduction	Comparison^a^	p	95% CI	
						Lower	Upper
Body-mass index, kg/m^2^				Insulin introduction—1 year after	0.070	− 1.57	0.04
Mean (sd)	28.4 (6.4)	29.3 (5.5)	29 (5.3)	Insulin introduction—2 years after	*0.013*	− 1.84	− 0.16
				1 year after–2 years after	> 0.999	− 1.09	0.62
Oral diabetes therapy				Insulin introduction–1 year after	*< 0.001*	8.0	27.0
n/Total (%)	98/99 (99)	72/87 (82.8)	67/82 (81.7)	Insulin introduction—2 years after	*< 0.001*	8.0	28.0
				1 year after–2 years after	> 0.999	− 12.00	14.00
Insulin daily dose, units/kg of body weight				Insulin introduction—1 year after	*< 0.001*	− 0.16	− 0.04
Mean (sd)	0.22 (0.12)	0.32 (0.24)	0.39 (0.26)	Insulin introduction—2 years after	*< 0.001*	− 0.23	− 0.10
				1 year after–2 years after	0.051	− 0.13	0.00
Glycated hemoglobin/A1c, %				Insulin introduction—1 year after	*< 0.001*	0.46	1.54
Mean (sd)	9.6 (2.1)	8.6 (2)	8.7 (1.7)	Insulin introduction—2 years after	*< 0.001*	0.39	1.49
				1 year after–2 years after	> 0.999	− 0.61	0.48
Diabetes controlled (adjusted A1c)				Insulin introduction—1 year after	*0.001*	− 37.0	− 8.0
n/total (%)	17/79 (21.5)	35/80 (43.8)	28/74 (37.8)	Insulin introduction—2 years after	*0.017*	− 31.0	− 2.0
				1 year after–2 years after	> 0.999	− 10.00	21.00
Total cholesterol, mg/dL				Insulin introduction—1 year after	*0.013*	2.51	28.06
Mean (sd)	201.7 (64.9)	188.1 (56.4)	180.6 (53.3)	Insulin introduction—2 years after	*< 0.001*	9.54	35.48
				1 year after–2 years after	0.521	− 5.48	19.93
LDL cholesterol, mg/dL							
Mean (sd)	103.8 (40.8)	104.7 (43.7)	100.5 (42.5)		0.425		
HDL cholesterol, mg/dL							
Mean (sd)	46.4 (13.3)	46 (13.5)	45.1 (11.4)		0.795		
Triglycerides, mg/dL				Insulin introduction—1 year after	0.175	− 8.19	70.08
Mean (sd)	201.8 (180.1)	174.4 (145.2)	160.9 (87.6)	Insulin introduction—2 years after	*0.023*	4.54	83.43
				1 year after–2 years after	> 0.999	− 26.00	52.08

GEE with normal distribution

^a^Bonferroni’s multiple comparisons

In a simple logistic regression analysis of the Group 2, we observed that the age, male gender and the presence of macroangiopathy were the main variables associated with achieving glycemic goals (individually adjusted A1c). In the multiple logistic regression analysis, only the age has influenced the diabetes control in this group. Each 1-year of age has increased 8% in the chance of achieving glycemic goals (p = 0.007) (Table 4).

**Table 4:** Logistic regression analysis—variables associated with achieving glycemic goals (individually adjusted A1c) during the first 2 years of insulin therapy follow up (Group 2)

Variable	Diabetes control		Total	OR_Crude_	95% CI		p	OR_Adjusted_	95% CI		p
	No	Yes			Lower	Upper			Lower	Upper	
Age, years				1.06	1.01	1.11	*0.018*	1.08	1.02	1.14	*0.007*
Mean (sd)	64.6 (8.5)	69.8 (10.8)	67.1 (10)								
Median (min.; max.)	65.5 (37; 76)	72.5 (45; 96)	67 (37; 96)								
Gender, n (%)											
Female	31 (64.6)	17 (35.4)	48	1.00							
Male	15 (37.5)	25 (62.5)	40	3.04	1.27	7.27	*0.012*				
Duration of diabetes, years				0.99	0.93	1.06	0.788				
Mean (sd)	17.5 (6)	17.1 (6.6)	17.3 (6.3)								
Median (min.; max.)	17 (7; 30)	16 (4; 33)	16.5 (4; 33)								
Duration of insulin use, years				0.99	0.93	1.06	0.825				
Mean (sd)	10.9 (6.4)	10.6 (6.5)	10.7 (6.4)								
Median (min.; max.)	10 (1; 24)	9 (2; 29)	10 (1; 29)								
Systemic arterial hypertension, n (%)											
No	3 (60)	2 (40)	5	1.00							
Yes	41 (51.2)	39 (48.8)	80	1.43	0.23	9.00	0.705				
Dyslipidemia, n (%)											
No	6 (75)	2 (25)	8	1.00							
Yes	38 (50.7)	37 (49.3)	75	2.92	0.55	15.41	0.206				
Neuropathy, n (%)											
No	37 (52.9)	33 (47.1)	70	1.00							
Yes	3 (60)	2 (40)	5	0.75	0.12	4.75	0.758				
Nephropathy, n (%)											
No	39 (60)	26 (40)	65	1.00							
Yes	0 (0)	8 (100)	8	#			#				
Retinopathy, n (%)											
No	37 (54.4)	31 (45.6)	68	1.00							
Yes	4 (57.1)	3 (42.9)	7	0.90	0.19	4.31	0.890				
Macroangiopathy, n (%)											
No	31 (60.8)	20 (39.2)	51	1.00							
Yes	9 (36)	16 (64)	25	2.76	1.02	7.43	*0.045*				
Insulin daily dose, units/kg of body weight				0.79	0.02	36.22	0.904				
Mean (sd)	0.22 (0.12)	0.21 (0.1)	0.21 (0.11)								
Median (min.; max.)	0.17 (0.04; 0.54)	0.18 (0.06; 0.42)	0.18 (0.04; 0.54)								

Logistic regression

# It’s not possible to estimate

## Discussion

The prevalence of clinical inertia during insulin therapy in a group of T2DM followed at a tertiary public Diabetes Center with limited pharmacologic armamentarium was between 56.2 and 65.8%. The association of human insulin with MET was the most frequent therapy and the occurrence of severe hypoglycemia was 3.1%. The BMI remained stable and the HDL cholesterol has increased. The main factors positively associated with individualized A1c targets were the age, male gender and the presence of retinopathy.

In the subgroup of T2DM we reviewed the first 2 years after insulin therapy introduction, the clinical inertia prevalence was 78.5% (at the moment of insulin therapy introduction), 56.2% (after 1 year) and 62.2% (after 2 years). The occurrence of severe hypoglycemia was 1%, the BMI has been stable, and the lipid profile has improved in the period. The available pharmacologic armamentarium was the same as Group 1 and the association of NPH insulin (once a day) with MET plus SU was the most frequent therapy. The main factor positively associated with individualized A1c targets during the period was the patient age.

The clinical inertia prevalence in both studied groups were similar to the literature findings [27, 28, 34, 35, 41]. Clinical inertia happens in both specialists and non-specialists (primary care physicians) follow up [35, 38, 39] and about only one-third of eligible patients for insulin treatment intensification had it done [16, 26, 27, 35, 38, 39]. The increasing of the age, the duration of diabetes, the presence of multiple diabetes chronic complications and the use of oral anti-hyperglycemic multiple therapy are the most reasons associated with a significant delay in the time to intensification [16, 27–30, 39, 40].

In our study, the patients had access only to MTF, SU and human insulin (NPH insulin and Regular) which are provided free of charge by the public healthcare system, the Brazilian Unified Health System (*Sistema Único de Saúde, SUS*). Insulin analogs and other antihyperglycemic therapies (GLP-1 analogues, DPP-IV inhibitors, Thiazolidinediones or SGLT2 Inhibitors) are not provided by the public healthcare system.

When we review insulin intensification studies [26, 27, 42, 51] and real-world basal insulin titration studies [43–45, 51] in T2DM patients, it seems that clinical inertia persists overtime and a growing body of evidence shows that there is often a disconnection between the setting and the achievement of treatment targets. Even with the increasing availability of effective glucose-lowering therapies, there is a failure to achieve established targets in almost half of people with diabetes [41].

The prevalence of severe hypoglycemia in our population was similar to the results of UKPDS and ADVANCE studies [46].

Most of our studied patients were overweight and the BMI remained stable from 1 year to another, with statistical differences only in the total period, following what literature shows about insulin therapy and its positive correlation with gaining weight [47]. The lipid profile improvements in our study were also similar to the literature findings [48–50].

It is kwon that intensification of pharmacotherapy requires glucose monitoring and medication adjustment at appropriate intervals when treatment goals are not achieved or maintained [12, 17, 18, 27–29, 40]. Another important aspect for the insulin therapy optimization and intensification is health literacy, defined as the ability to obtain, read, understand and use healthcare information to make appropriate health decisions and to follow instructions for treatment [19–23].

One of the limitations of our study is that we did not measure health literacy, health numeracy or patient adherence to medications [24, 25, 29, 30]. On the other hand, the strengths of our study were: (1) The glycemic goals were individualized in according to clinical conditions and comorbidities of the patient and (2) It was possible to perform the analysis of different moments of insulin therapy.

The discussion about clinical inertia and the difficulties of achieving the glycemic targets range from the causes related to the healthcare professionals [14–16, 26–28, 41], the disease [10, 34], the patients [17–30] and the healthcare system [27, 28].

## Conclusions

We can conclude that in a population of T2DM patients ongoing insulin therapy, followed at a tertiary public Diabetes Center from an upper-middle income country with limited pharmacologic armamentarium, the prevalence of clinical inertia ranged from 56.2 to 78.5% at different moments of the insulin therapy (first 2 years after the introduction and long term).

## Additional files

**Additional file 1: Figure S1.** A1c subgroup analyses (Group 1).

**Additional file 2: Figure S2.** A1c subgroup analyses (Group 2).

## Abbreviations

AACE: American Association of Clinical Endocrinologists
ACE: American College of Endocrinology
ADA: American Diabetes Association
AHT: anti hyperglycemic therapy
A1C: glycated hemoglobin
BMI: body mass index
CIs: 95% confidence intervals
DPP-IV inhibitors: dipeptidyl peptidase IV inhibitors
DM: diabetes mellitus
EASD: European Association for the Study of Diabetes
GEE: Generalized Estimating Equations
GLP-1 analogues: glucose like peptide 1 analogues
HDL: high-density lipoprotein cholesterol
HPLC: high-performance liquid Chromatography
IDF: International Diabetes Federation
IU: International Units
kg: kilogram
LADA: Latent Autoimmune Diabetes of the Adult
LDL: low-density lipoprotein cholesterol
m^2^: square meters
MET: Metformin
mg/dL: milligram per deciliter
mmol/L: millimoles per liter
MODY: maturity-onset diabetes of the young
n: number
ns: not statistically significant
nv: normal value
OAD: oral antidiabetes drugs
OR: odds ratio
P: probability
SD: standard deviation
SGLT2 Inhibitors: sodium-glucose co-transporter 2 inhibitors
SU: sulphonylurea
SUS: Sistema Único de Saúde (Unified Health System)
TC: total cholesterol
TG: triglycerides
T1DM: type 1 diabetes mellitus
T2DM: type 2 diabetes mellitus
UKPDS: United Kingdom Prospective Diabetes Study
Yr: year
