# Supplementary figures and images for: Clinical inertia on insulin treatment intensification in type 2 diabetes mellitus patients of a tertiary public diabetes center with limited pharmacologic armamentarium from an upper-middle income country
Source: Diabetol Metab Syndr. 2018 Oct 29;10:77. doi: 10.1186/s13098-018-0382-x (PMC6206856; doi:10.1186/s13098-018-0382-x)

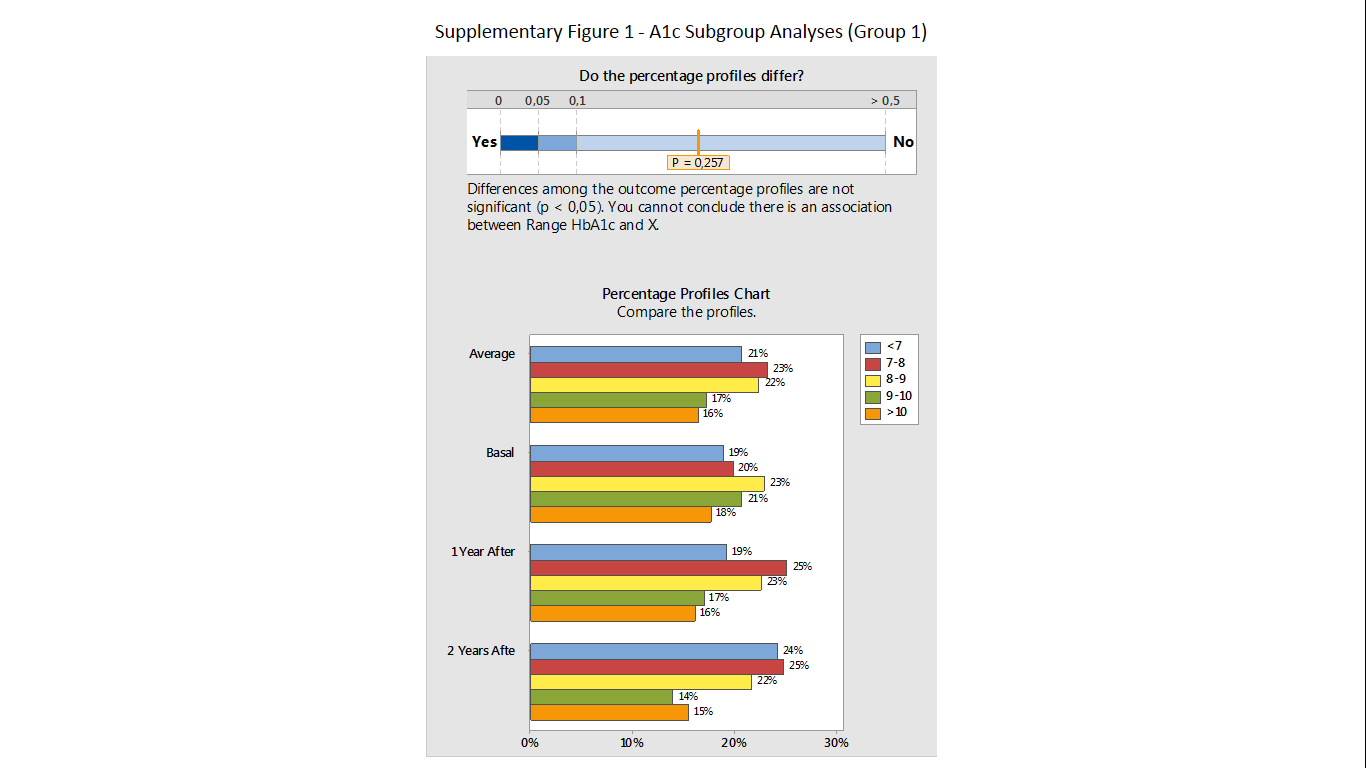

Supplement: Supplementary file 1 — Additional file 1: Figure S1. A1c subgroup analyses (Group 1). [file 13098_2018_382_MOESM1_ESM.png]

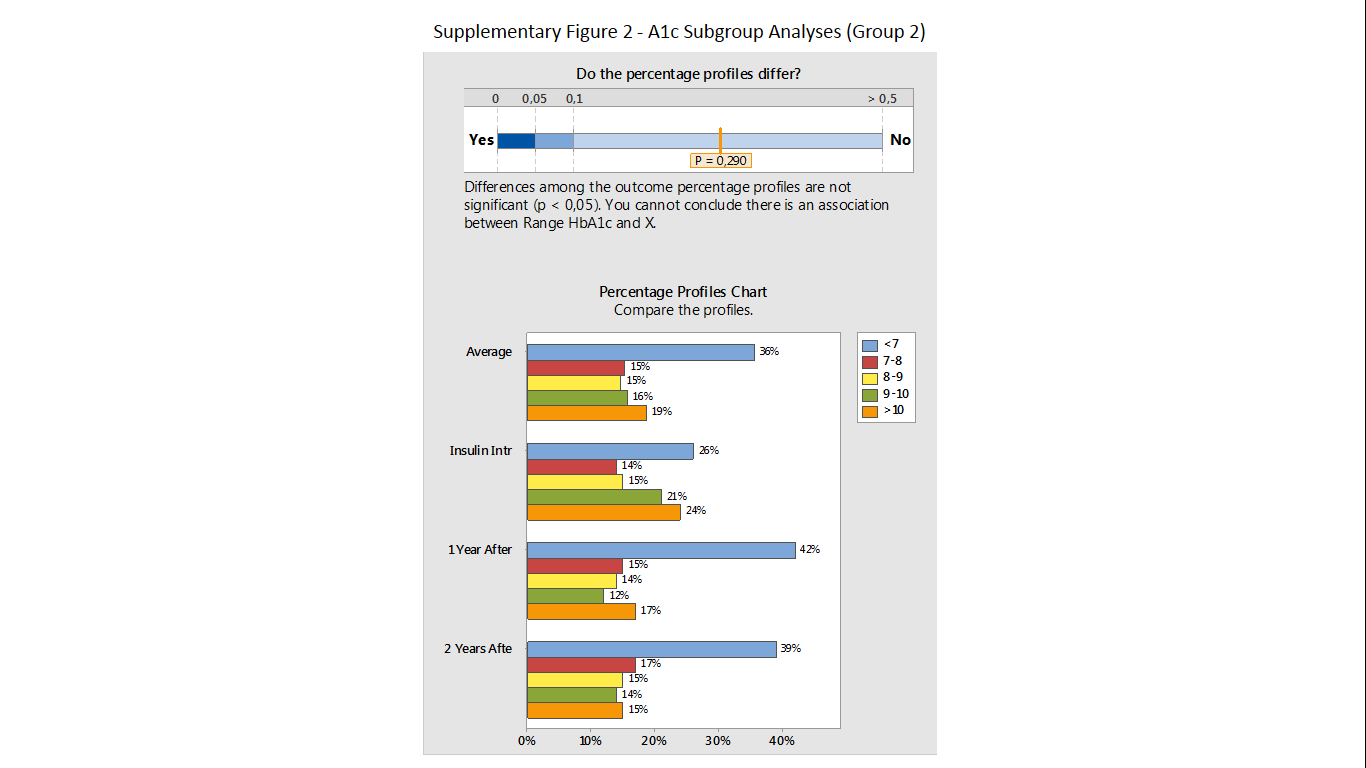

Supplement: Supplementary file 2 — Additional file 2: Figure S2. A1c subgroup analyses (Group 2). [file 13098_2018_382_MOESM2_ESM.png]
